# Inflammasome activation and accelerated immune aging in autoimmune disorders

**DOI:** 10.3389/fragi.2025.1688060

**Published:** 2025-09-30

**Authors:** Rahul Mittal, Danay Saavedra, Mannat Mittal, Joana R. N. Lemos, Khemraj Hirani

**Affiliations:** ^1^ Diabetes Research Institute, University of Miami Miller School of Medicine, Miami, Florida, FL, United States; ^2^ Division of Endocrinology, Diabetes, and Metabolism, Department of Medicine, University of Miami Miller School of Medicine, Miami, Florida, FL, United States

**Keywords:** cellular senescence, senescence-associated secretory phenotype (SASP), inflammaging, immune senescence, autoimmunity

## Abstract

Autoimmune diseases, particularly those with early onset such as systemic lupus erythematosus, juvenile idiopathic arthritis, and type 1 diabetes, are paradoxically characterized by molecular and cellular features typically associated with aging. These include telomere shortening, mitochondrial dysfunction, epigenetic alterations, and skewed immune cell phenotypes, which are considered hallmarks of immunosenescence. This perspective explores the hypothesis that aberrant inflammasome activation, particularly of the NLRP3 complex, serves as a key upstream driver of premature immune aging in autoimmunity. We examine how chronic inflammasome signaling induces senescence through pro-inflammatory cytokine production and oxidative stress, reinforces the senescence-associated secretory phenotype (SASP), and perpetuates immune dysregulation. By reframing autoimmunity as a disorder of accelerated immune aging, we highlight emerging opportunities for therapeutic intervention using senolytics, inflammasome inhibitors, and lifestyle modifications. In addition, incorporating biomarkers of immune aging into clinical assessment may enable precision immunogerontology, particularly in pediatric populations where biological and chronological age may be dissociated. Elucidating the relationship between inflammasome signaling and immune senescence provides a critical framework for understanding autoimmune pathogenesis and for developing interventions that modify disease course by targeting age-associated mechanisms.

## 1 Introduction

Autoimmune diseases are commonly characterized by a breakdown in immune tolerance and persistent activation of inflammatory pathways (Hoi et al., 2024; DiMeglio et al., 2018; Siegel and Sammaritano, 2024; Quattrin et al., 2023; Mittal et al., 2024; Mittal et al., 2025). Although these conditions can affect individuals across the lifespan, a significant proportion of cases, including systemic lupus erythematosus (SLE), juvenile idiopathic arthritis (JIA), and type 1 diabetes (T1D), begin early in life (Pan et al., 2023; Boteanu et al., 2025). This early onset is paradoxical, as affected individuals often exhibit molecular and cellular features typically associated with an aged immune system (van den Hoogen et al., 2015; Tsai et al., 2019; Handono et al., 2021; Mayerl and Prelog, 2012; Shapiro et al., 2023).

**FIGURE 1:**
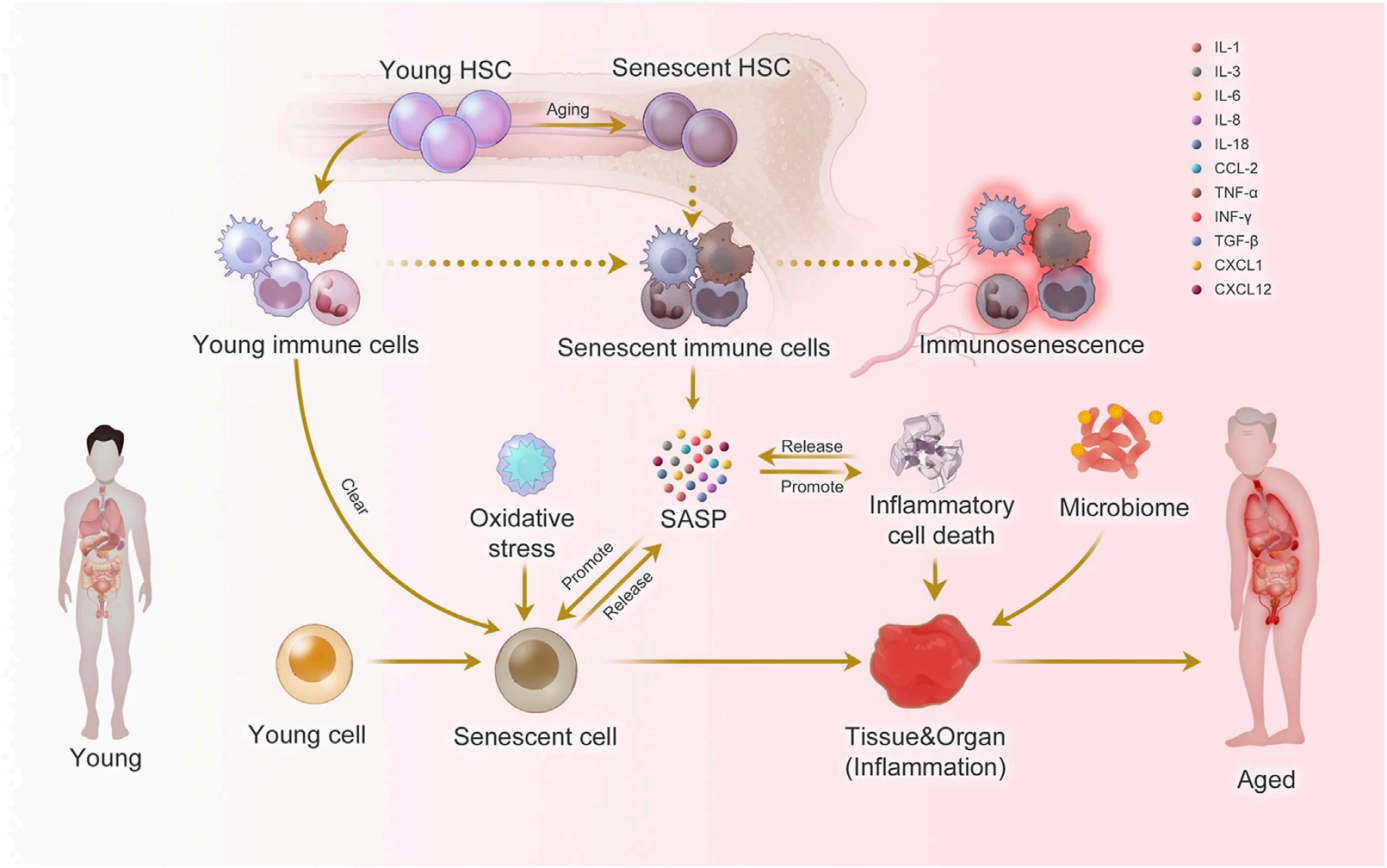
Inflammaging at the molecular, cellular, and organ levels. During the aging process, almost all cells in the body undergo senescence, a state characterized by a dysfunctional state and senescence-associated secretory phenotype (SASP). While immune cells play a crucial role in recognizing and eliminating these senescent cells, they are also affected by SASP, leading to a phenomenon called immunosenescence. Immunosenescence can impair the immunity to respond to infections and diseases, making the organism more vulnerable to illnesses. Moreover, the accumulation of senescent cells can trigger inflammation in organs, leading to organ damage and an increased risk of age-related diseases. This process is exacerbated by positive feedback loops that drive the accumulation of inflammation and organ damage, leading to further inflammation and an even higher risk of aging-related diseases. Taken from (Li X. et al., 2023) under a Creative Commons Attribution 4.0 International License (http://creativecommons.org/licenses/by/4.0/).

In physiological aging, the immune system undergoes a process known as immunosenescence, which encompasses several functional and structural alterations. These include reduced thymic output of naïve T cells, accumulation of terminally differentiated and exhausted lymphocytes, impaired antigen responsiveness, and a chronic low-grade inflammatory state referred to as “inflammaging” (Figure 1) (Teissier et al., 2022; Liao et al., 2022; Li X. et al., 2023). Surprisingly, many of these hallmarks are also observed in young individuals with autoimmune disease. Pediatric and adolescent patients with SLE have been shown to exhibit shortened telomeres, mitochondrial dysfunction, epigenetic alterations, and elevated expression of cell cycle inhibitors such as p16^INK4a^ and p21^CIP1^ (Tsai et al., 2019; Melk et al., 2005; Yang et al., 2018; Gao et al., 2019; Kurosaka et al., 2006; Haque et al., 2013). Additionally, immune cell compartments in these individuals often display skewing toward memory and senescent phenotypes, resembling the profile seen in elderly populations (Li et al., 2022; Tan et al., 2024; Rother and van der Vlag, 2015; Kalim et al., 2021). Functionally, immune senescence results in impaired immune surveillance, reduced vaccine efficacy, and heightened susceptibility to infections (Allen et al., 2020). Importantly, senescent cells, despite being metabolically active, are often dysfunctional, contributing to tissue damage and perpetuating systemic inflammation (Tripathi et al., 2021; Palmer et al., 2022).

**FIGURE 2:**
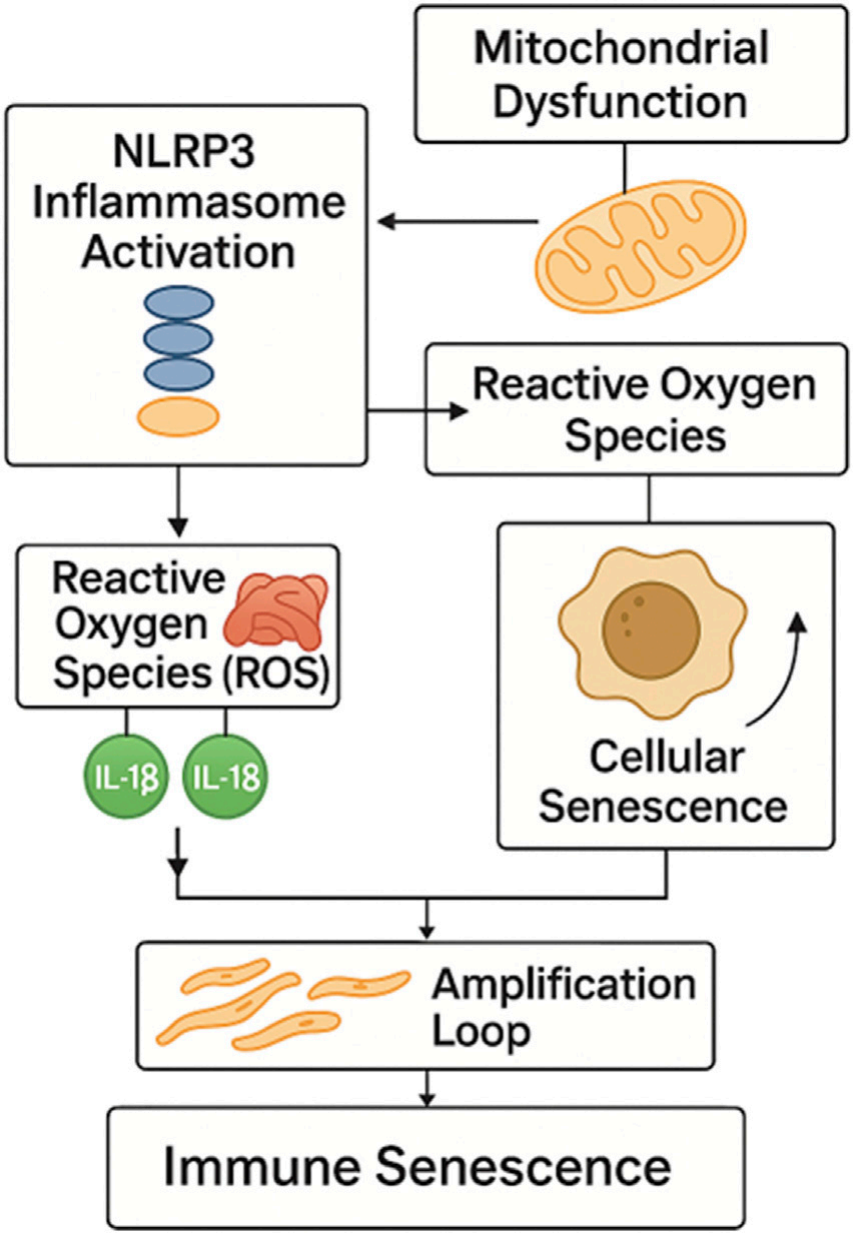
NLRP3 inflammasome-driven pathway to immune senescence. Activation of the NLRP3 inflammasome induces mitochondrial dysfunction and ROS generation, leading to IL-1β and IL-18 release. These cytokines promote cellular senescence and SASP amplification, establishing a feed-forward loop that drives premature immune aging in autoimmunity. NLRP3 - NOD-like receptor family pyrin domain containing 3; ROS - Reactive Oxygen Species; IL-1β - Interleukin-1 beta; IL-18 - Interleukin-18; SASP - Senescence-Associated Secretory P.henotype.

These observations suggest that premature immune aging may be a fundamental feature of autoimmunity, rather than a secondary consequence of chronic inflammation or immunosuppressive therapy. However, the mechanistic basis for this accelerated senescence remains incompletely understood. Among the potential upstream drivers is aberrant activation of the inflammasome complex, particularly the NLRP3 inflammasome (Figure 2) (Kelley et al., 2019; Paik et al., 2021; Seoane et al., 2020). Inflammasome activation results in the maturation and secretion of interleukin-1β (IL-1β) and interleukin-18 (IL-18), cytokines known to induce oxidative stress, DNA damage, and pro-senescent signaling pathways in both immune and non-immune cells (Yao et al., 2024; Li H. et al., 2023).

Despite advances in immunosenescence research, direct, within-patient evidence linking NLRP3 inflammasome activation with cellular senescence in autoimmune diseases remains limited. Most available data do not derive from paired analyses that simultaneously assess inflammasome activation and senescence markers within the same cellular populations or across the same individuals. Instead, the proposed link between NLRP3 activity and senescence in human autoimmunity is largely inferred. Current understanding is primarily based on converging but separate lines of evidence, such as animal models, mechanistic cell-based studies, and independent clinical observations of senescence-associated signatures and inflammasome activity. As a result, the hypothesized NLRP3–senescence axis remains a compelling but as yet unproven framework in the context of robust human clinical cohorts, highlighting the need for further longitudinal and mechanistically integrated studies to establish causality.

This perspective article aims to explore the hypothesis that inflammasome activation may act as a central mechanism linking autoimmunity to accelerated immune aging. We synthetize current evidence linking chronic inflammasome signaling to the emergence of senescence-associated phenotypes in young patients, emphasizing mechanistic pathways and feedback loops that sustain immune dysfunction. Furthermore, we discuss the implications of this relationship for understanding disease pathogenesis and for developing therapeutic strategies that target the intersection of inflammation and immune aging, with particular attention to early-onset autoimmunity.

## 2 Inflammasomes as accelerators of immune senescence

### 2.1 Mechanistic link

Inflammasomes are cytosolic multiprotein complexes that serve as innate immune sensors, detecting pathogen-associated molecular patterns and damage-associated molecular patterns. Among them, the NLRP3 inflammasome is the most extensively studied and is activated by a wide range of stimuli, including mitochondrial dysfunction, extracellular ATP, crystalline substances, and various environmental stressors (Kelley et al., 2019; Paik et al., 2021; Seoane et al., 2020).

Upon activation, the NLRP3 inflammasome facilitates the cleavage of pro–caspase-1 into its active form, caspase-1 (Kelley et al., 2019; Paik et al., 2021; Seoane et al., 2020). Active caspase-1 subsequently processes pro–interleukin-1β (IL-1β) and pro–interleukin-18 (IL-18) into their mature, secreted forms (Kelley et al., 2019; Paik et al., 2021; Seoane et al., 2020). Sustained production of IL-1β and IL-18 results in chronic inflammatory signaling, which promotes the generation of reactive oxygen species (ROS), loss of mitochondrial membrane potential, and accumulation of nuclear DNA damage (Harijith et al., 2014; Patergnani et al., 2021; Hong et al., 2024; Checa and Aran, 2020). These intracellular stressors contribute directly to the induction of cellular senescence pathways, including the activation of p53/p21 and p16/Rb signaling cascades, which enforce irreversible cell cycle arrest.

Mitochondrial dysfunction, a central feature of senescent cells, also serves to propagate inflammasome activity (Wei et al., 2025; Vasileiou et al., 2019; Picca et al., 2017). Oxidized mitochondrial DNA released into the cytosol can further stimulate NLRP3, thereby creating a feed-forward loop that perpetuates inflammasome activation and inflammatory cytokine production (Zhang X. et al., 2025). This bidirectional relationship establishes a cellular environment highly conducive to senescence induction, particularly within immune and barrier tissue compartments.

Pharmacological or genetic inhibition of NLRP3 or downstream cytokine pathways has been shown to attenuate these senescence-associated features in multiple experimental models (Marín-Aguilar et al., 2020; Cañadas-Lozano et al., 2020; Navarro-Pando et al., 2021; Quijano et al., 2022). These findings suggest a causal role for inflammasome signaling in promoting and maintaining the senescent phenotype.

### 2.2 SASP and feedback loops

Senescent cells develop a complex pro-inflammatory secretome known as the senescence-associated secretory phenotype, or SASP (Wang B. et al., 2024; Kuma et al., 2021; Roger et al., 2021). This secretory profile includes a broad spectrum of cytokines, chemokines, matrix metalloproteinases, and growth factors, many of which are directly or indirectly regulated by IL-1β signaling (Lopes-Paciencia et al., 2019). Inflammasome activation amplifies the SASP through autocrine and paracrine mechanisms (Yue et al., 2022). IL-1β produced via inflammasome signaling activates NF-κB and C/EBPβ transcriptional programs, which in turn promote the expression of additional SASP components such as IL-6 and IL-8 (Kelley et al., 2019; Paik et al., 2021; Seoane et al., 2020). This creates a self-reinforcing loop in which inflammasome-derived cytokines potentiate further SASP production and sustain the senescent state (Fu et al., 2025). SASP factors exert significant bystander effects, as they can induce DNA damage, oxidative stress, and inflammatory signaling in neighboring cells, thereby spreading senescence beyond the initially affected population (Han et al., 2024; Ohtani, 2022). This propagation of the senescent phenotype contributes to tissue dysfunction and systemic inflammation, hallmarks of immune aging.

In immune cells, the persistent activation of inflammasomes and sustained exposure to SASP components alter cell fate and function (Kuma et al., 2021; Sebastian-Valverde and Pasinetti, 2020; Latz and Duewell, 2018; Meyers and Zhu, 2020; Muela-Zarzuela et al., 2024). Naïve and memory T-cell pools become skewed toward terminal differentiation and exhaustion, B cells exhibit reduced antigen specificity and increased autoreactivity, and myeloid cells adopt a pro-inflammatory, senescent-like phenotype (Gritsenko et al., 2020; Liu et al., 2023; Zheng et al., 2023; Zhao et al., 2022; Camell et al., 2019; Ma et al., 2019; Wang L. et al., 2024; Zhu et al., 2023; Behmoaras and Gil, 2021; Hu et al., 2019). Within the stromal and epithelial compartments, SASP and inflammasome activity disrupt tissue architecture and impair regenerative capacity (Wan et al., 2021; Paez-Ribes et al., 2019).

Together, these mechanisms illustrate how inflammasome activation not only induces cellular senescence but also stabilizes and expands it through SASP amplification and inflammatory feedback. The integration of these pathways is a central feature of accelerated immune aging in the context of chronic inflammation and autoimmunity.

## 3 Early-onset inflammaging in autoimmunity

The term “inflammaging” refers to a chronic, low-grade, sterile inflammatory state that gradually develops with advancing age and is considered a hallmark of immunosenescence (Franceschi et al., 2018; Fulop et al., 2023; Franceschi and Campisi, 2014). It is characterized by persistent elevation of pro-inflammatory mediators such as interleukin-1β (IL-1β), interleukin-6 (IL-6), tumor necrosis factor-alpha (TNF-α), and C-reactive protein (CRP), occurring in the absence of overt infection (Fülöp et al., 2019). This inflammatory environment is sustained by lifelong exposure to antigenic stimuli, cumulative tissue damage, impaired autophagy, mitochondrial dysfunction, and the accumulation of senescent cells that secrete pro-inflammatory factors (Ajoolabady et al., 2024).

Although inflammaging was originally conceptualized in the context of geriatric biology, emerging evidence indicates that many of its immunologic, molecular, and metabolic features are present in young individuals with autoimmune diseases (Franceschi et al., 2025). Early-onset autoimmune conditions such as SLE, JIA, and T1D display an inflammatory milieu that phenocopies the immune landscape of aged individuals (Frazzei et al., 2022; Goronzy and Weyand, 2012). In these patients, inflammatory biomarkers remain elevated even during periods of clinical remission, suggesting a constitutive and potentially irreversible rewiring of immune regulation toward a pro-inflammatory state. This early-onset inflammaging is not merely a reflection of disease activity but may instead constitute a foundational process driving disease pathogenesis and progression.

### 3.1 Immune cell rewiring

One of the central consequences of early inflammaging in autoimmunity is the reprogramming of immune cell lineages toward pro-inflammatory, senescent-like phenotypes (Alexander et al., 2024). Chronic activation of inflammasome complexes, particularly NLRP3, promotes sustained production of IL-1β and IL-18, which act on both adaptive and innate immune compartments (Kelley et al., 2019). In T cells, prolonged exposure to inflammatory cytokines alters differentiation trajectories, favoring the expansion of effector memory (Tem) and terminally differentiated effector memory RA (TEMRA) populations at the expense of the naïve T-cell pool (Kim and Harty, 2014; Raeber et al., 2018; Fritsch et al., 2006; Skapenko et al., 1999). These terminal subsets exhibit reduced proliferative potential, shortened telomeres, impaired synapse formation, and a biased cytokine profile skewed toward interferon-gamma and cytotoxic granule production (Ohmes et al., 2022).

CD8^+^ T cells lacking the co-stimulatory molecule CD28, a well-established marker of replicative senescence, are frequently expanded in young autoimmune patients and contribute to tissue damage through cytotoxic and inflammatory mechanisms (Żabińska et al., 2016; Minning et al., 2019). Similarly, CD4^+^ T cells demonstrate increased expression of PD-1 and other exhaustion markers, consistent with chronic antigen stimulation and senescence-associated dysfunction (Fischer et al., 2022; Saggau et al., 2024; Koutsonikoli et al., 2024).

B cells are also affected by this rewiring. Chronic inflammatory signaling, particularly via IL-1β and IL-6, disrupts central and peripheral tolerance checkpoints, promoting the survival of autoreactive clones (Maeda et al., 2010; Nicholas et al., 2025; Canny and Jackson, 2021; Arkatkar et al., 2017; Nagafuchi et al., 1993). These B cells often acquire a hyperactive phenotype, with increased spontaneous antibody production, elevated class switching, and loss of regulatory B-cell subsets (Iwata et al., 2023). In parallel, age-associated B cells (ABCs), a population initially characterized in aged mice and humans, are found at higher frequencies in young individuals with autoimmune disease (Mouat et al., 2022; Li Z. Y. et al., 2023; Wehr et al., 2004; Cancro, 2020; Xie et al., 2025; Nickerson et al., 2023; Su et al., 2025; Miranda-Prieto et al., 2025; Sachinidis et al., 2025). These ABCs exhibit pro-inflammatory characteristics and are potent antigen-presenting cells, further perpetuating autoreactivity (Ratliff et al., 2013; Riley et al., 2017; Qin et al., 2022).

### 3.2 Epigenetic and metabolic signatures

In addition to phenotypic and functional changes, early-onset inflammaging in autoimmunity is accompanied by alterations in the epigenetic and metabolic landscapes of immune cells (Nardini et al., 2018). Epigenetic aging, assessed through DNA methylation clocks, has been shown to be accelerated in patients with autoimmune diseases (Somers et al., 2024; Pérez et al., 2020). These findings suggest that chronic immune activation may impose an age-related epigenetic program on hematopoietic and immune progenitors. Methylation changes are particularly enriched in promoters of genes involved in cytokine signaling, antigen presentation, and lymphocyte differentiation (Morales-Nebreda et al., 2019; Calle-Fabregat et al., 2020). These modifications may serve as both biomarkers and mediators of inflammaging, reinforcing transcriptional programs that sustain inflammatory signaling and senescence.

Metabolic reprogramming is another key feature of early inflammaging. Immune cells from autoimmune patients often shift from oxidative phosphorylation to lactate-producing glycolysis, even in the presence of oxygen, a phenomenon known as the Warburg effect (Granados et al., 2017; Mocholi et al., 2025; Takeshima et al., 2019). This shift is typically associated with activation and effector differentiation, but when sustained, it contributes to mitochondrial dysfunction, excessive ROS production, and metabolic stress (Palmer et al., 2015; Yennemadi et al., 2023; Gergely et al., 2002). Furthermore, impaired mitophagy and accumulation of dysfunctional mitochondria serve as endogenous activators of inflammasomes, creating a positive feedback loop between metabolic stress and inflammatory signaling (Yuk et al., 2020; Mishra et al., 2021; Kim et al., 2016; Liu et al., 2018).

Together, these epigenetic and metabolic signatures reflect a biologically aged immune phenotype in individuals with early-onset autoimmunity. Importantly, they support the hypothesis that immune aging processes, once thought to be restricted to the elderly, are operational much earlier in life in the context of chronic auto-inflammatory disease.

## 4 Clinical and translational implications

Conventional clinical paradigms often use chronological age as a primary determinant of immune status, disease prognosis, and therapeutic strategy. However, in the context of autoimmune diseases that manifest during childhood or early adulthood, this approach may be inadequate. Evidence of premature immune senescence in young patients suggests that biological age, rather than chronological age, may be a more accurate indicator of disease severity, immunologic competence, and long-term outcomes. Recognition of this discrepancy has important implications for personalized medicine, as it calls for an age-stratified model based not solely on calendar age but also on molecular and cellular indicators of immune aging. Integrating biomarkers of senescence and inflammaging into clinical practice could enable earlier identification of high-risk patients and guide the selection of targeted interventions aimed at modulating the underlying age-related mechanisms contributing to autoimmunity.

### 4.1 Therapeutic targeting opportunities

The identification of senescence and inflammasome activation as key drivers of early-onset autoimmunity opens new avenues for therapeutic intervention (Supplementary Table 1). One promising approach involves the use of senolytic agents, which selectively eliminate senescent cells, and SASP modulators, which suppress the deleterious secretory phenotype associated with these cells (Shao et al., 2021; Chen et al., 2025; Atlante et al., 2025; Zhang M. et al., 2025; Kim et al., 2025). Although originally developed for geriatric applications, these compounds may have utility in younger patients with autoimmune disease who exhibit premature immune aging.

Another class of therapeutic agents with potential relevance is inflammasome inhibitors. Pharmacological blockade of the NLRP3 inflammasome or its downstream effectors, such as interleukin-1β and interleukin-18, has demonstrated efficacy in preclinical models of autoimmune and inflammatory disease. These agents may act not only to suppress acute inflammation but also to decelerate immune aging processes by disrupting the feed-forward loops that maintain chronic inflammasome activation and cellular senescence.

In addition to pharmacologic strategies, lifestyle-based interventions may offer adjunctive benefit by modulating immune aging. Regular physical activity, dietary interventions, and caloric restriction mimetics have been shown to attenuate systemic inflammation and support mitochondrial function (Abad-Jiménez et al., 2024; Vitetta and Anton, 2007; Gabandé-Rodríguez et al., 2019; Kyriazis et al., 2022). When combined with molecularly targeted therapies, these interventions may produce synergistic effects, improving immune resilience and altering disease trajectory in individuals with early signs of immune senescence.

While the targeting of senescent cells holds promise for mitigating age-related dysfunction and chronic inflammation, it is important to recognize potential risks. Senescent cells may also play context dependent roles in tissue repair, remodeling, and the maintenance of homeostasis (Faust et al., 2020; Gadecka et al., 2025; Demaria et al., 2014). Broad elimination or functional modulation of these cells could therefore disrupt beneficial processes, impair regenerative capacity, or compromise immune surveillance. These considerations highlight the need for approaches that achieve sufficient selectivity, whether by context, timing, or cell type, to minimize unintended consequences while preserving physiological functions.

### 4.2 Biomarker development for risk stratification and monitoring

The integration of biomarkers that reflect immune aging into clinical assessment could transform the management of autoimmune disease (Supplementary Table 2). Telomere length, expression of senescence-associated genes such as p16^INK4a^ and p21^CIP1^, and levels of SASP components including IL-6, IL-1β, and matrix metalloproteinases offer potential as diagnostic and prognostic tools. Additionally, quantification of inflammasome components, including NLRP3 expression and caspase-1 activity, may provide insight into the inflammatory status of immune cells and their propensity to enter senescence.

These biomarkers could be employed to stratify patients based on biological age and inflammaging burden, enabling more precise prediction of disease course and responsiveness to immune-modifying therapies. Longitudinal monitoring of these parameters could also help assess therapeutic efficacy, particularly in interventions aimed at mitigating immune senescence or targeting the inflammasome-senescence axis.

### 4.3 Age-specific considerations

Age-specific considerations are critical when evaluating inflammasome-targeted therapies, as efficacy and safety profiles established in adults cannot be assumed to directly translate to pediatric populations. Importantly, therapeutic targeting of the inflammasome pathway is already feasible in pediatric autoimmunity. IL-1 blockers, such as anakinra, have demonstrated efficacy and safety in children with systemic juvenile idiopathic arthritis, including robust long-term data (Quartier et al., 2011; Giancane et al., 2022) Canakinumab has induced sustained remissions in pediatric CAPS with favorable multi-year safety outcomes (Kuemmerle-Deschner et al., 2011; Brogan et al., 2019). By contrast, direct oral NLRP3 inhibitors (dapansutrile/OLT1177) remain limited to adult clinical data in conditions such as gout and heart failure (Klughammer et al., 2023; Terkeltaub et al., 2019; Wohlford et al., 2020). Thus, pediatric translation of such agents will require dedicated studies addressing dosing, growth, and infection risk.

To date, no clinical trials have evaluated senolytic therapies such as dasatinib plus quercetin or fisetin in pediatric populations for the management of immune aging or autoimmunity. Cellular senescence contributes to developmental processes, tissue repair, and neurodevelopment, while preservation of vaccine responsiveness and normal growth remains critical in children. These considerations argue against indiscriminate senescent cell clearance in this age group. Accordingly, senescence-targeted interventions should be restricted to rigorously controlled research settings. Within such contexts, current best practice favors reversible modulation of SASP rather than broad elimination of senescent cells.

## 5 Future directions

### 5.1 Integrated profiling of immune senescence and inflammasome activity

A major priority for advancing the understanding of early-onset autoimmunity involves the implementation of longitudinal studies that systematically characterize immune aging across disease progression. These studies should incorporate multi-parametric immune phenotyping, quantitative assessment of senescence-associated biomarkers, and detailed profiling of inflammasome activity. Pediatric and adolescent populations represent a critical cohort for such investigations, as early disease stages may reveal mechanistic insights into the initial triggers of immune senescence and chronic inflammation. The integration of single-cell transcriptomics, epigenetic landscape mapping, proteomics, and metabolomics will provide a comprehensive framework to define immune trajectories and identify inflection points predictive of disease acceleration or remission.

By correlating molecular signatures with clinical phenotypes, it may become possible to identify distinct biological aging patterns that underlie disease heterogeneity, inform therapeutic response, and facilitate the development of predictive tools for early intervention.

### 5.2 Toward precision immunogerontology

The concept of precision immunogerontology emphasizes the need to tailor interventions based on biological immune age rather than relying solely on chronological metrics. In the context of autoimmune disease, this approach requires stratification of patients according to validated biomarkers of immune senescence, inflammaging intensity, and functional immune reserve. Personalized immunogerontologic strategies could involve dynamic modulation of senescence-associated signaling pathways, selective depletion of senescent cells, or restoration of mitochondrial and epigenetic integrity in immune progenitors.

Advancing this framework will require the development of clinical algorithms that integrate molecular diagnostics with real-time immune monitoring. These tools will support individualized treatment decisions and enable risk-adjusted strategies to prevent disease progression or recurrence. Ultimately, precision immunogerontology may redefine therapeutic windows and optimize long-term outcomes for patients with early-onset autoimmune disorders.

### 5.3 Bridging the fields of immunology and aging research

The intersection between immunology and aging biology remains underexplored, particularly in the setting of autoimmunity. Promoting interdisciplinary collaboration between researchers in cellular senescence, mitochondrial biology, epigenetics, and immune regulation is essential to uncover convergent mechanisms that drive immune dysfunction across the lifespan. Shared platforms for data integration and cross-disciplinary communication will accelerate the translation of basic science discoveries into therapeutic applications.

Additionally, fostering collaborative consortia that include pediatric rheumatologists, geroscientists, computational biologists, and clinical trialists will enhance the design of studies that address the complex interplay between immune development, senescence, and inflammation. Such efforts will be crucial to identify early biomarkers of immune aging, establish causal relationships between inflammasome activity and senescence, and validate age-modifying interventions for autoimmune disease.

## 6 Discussion

The convergence of autoimmunity and premature immune aging represents a paradigm shift in our understanding of disease initiation and progression in young patients. While traditionally framed as pathologies of immune hyperactivity, autoimmune diseases are increasingly recognized to embody features of immune senescence typically reserved for advanced age. The co-occurrence of telomere attrition, diminished thymic output, mitochondrial dysfunction, and a proinflammatory cytokine milieu in young autoimmune cohorts highlight a biologically aged immune landscape occurring decades earlier than anticipated. At the center of this shift lies inflammasome biology. The NLRP3 inflammasome, in particular, emerges not merely as a mediator of acute inflammation but as a pivotal orchestrator of immune aging. Its activation instigates a cascade of intracellular events including mitochondrial impairment, reactive oxygen species accumulation, and DNA instability that converge on classical senescence pathways. The downstream elaboration of interleukin 1 beta and interleukin 18 consolidates this trajectory by reinforcing the senescence associated secretory phenotype, which extends proaging signals across tissue compartments through paracrine amplification. Thus, inflammasome signaling constitutes both a precipitant and perpetuator of immune dysfunction.

Bidirectional interactions between mitochondrial dysfunction and NLRP3 inflammasome activation are now well-established (Qiu et al., 2022; Pan et al., 2018; Billingham et al., 2022; Bronner et al., 2015; He et al., 2025; Ning et al., 2025; Wu et al., 2025; Huang et al., 2025). Damaged or oxidized mitochondrial DNA (ox-mtDNA) released into the cytosol activates NLRP3, while inhibition of mitophagy or augmented mitochondrial ROS amplifies inflammasome assembly and IL-1β secretion (Xian et al., 2025; Shimada et al., 2012; Zhong et al., 2018; Nakahira et al., 2011; Zhou et al., 2011; Iyer et al., 2013; Heid et al., 2013). Studies have shown that ox-mtDNA can exit via mitochondrial permeability transition pore (mPTP) or VDAC channels, activating both NLRP3 and interferon responses (Xian et al., 2022; Kim et al., 2019; Baik et al., 2023). Conversely, inflammasome activation can exacerbate mitochondrial injury (Yu et al., 2014). Gasdermin-D, downstream of inflammasome activation, forms pores that compromise mitochondrial integrity, contributing to mitochondrial dysfunction during pyroptosis (Miao et al., 2023; Tang et al., 2024; de Torre-Minguela et al., 2021). Thus, a feed-forward loop exists in which mitochondrial stress primes NLRP3 activation, and inflammasome-mediated pyroptotic processes in turn worsen mitochondrial health, consistent with mechanisms underlying “inflammaging” in autoimmunity.

Crucially, the immunosenescent profile observed in youth is not merely epiphenomenal. Mounting data implicates these cellular alterations in the etiology of autoimmune pathogenesis itself. The persistence of senescent T and B cell subsets, impaired tolerance checkpoints, and the emergence of proinflammatory myeloid phenotypes reflect a systemic remodeling of immunity that precedes and sustains autoimmunity. In this light, biological aging of the immune system is not an outcome but a driver of disease. This framework mandates a reassessment of how age is conceptualized in autoimmune disease. Chronological age is no longer a sufficient proxy for immune competence or resilience. Instead, biologically grounded metrics including markers of cellular senescence, inflammasome activity, and epigenetic age offer a more precise approach through which to interpret disease severity, progression, and therapeutic responsiveness.

Therapeutic implications are equally profound. Targeting the inflammasome senescence axis may yield interventions that not only suppress inflammation but recalibrate immune aging itself. Agents that abrogate inflammasome activation, modulate senescence associated secretory activity, or selectively eliminate senescent immune populations represent rational strategies for disrupting the self-amplifying cycle of immune decay. When complemented by metabolic and lifestyle interventions aimed at restoring mitochondrial function and reducing oxidative load, these strategies offer a multifaceted approach to restoring immune homeostasis. Future studies should focus on delineating the temporal sequence linking inflammasome activity with senescence onset in autoimmunity. Disentangling cause from consequence will require longitudinal designs and integrated omics profiling, particularly in early disease stages. Collaborative efforts spanning immunology, geroscience, and systems biology will be essential to operationalize biological age as a clinically actionable variable.

Reframing autoimmunity as a disorder of accelerated immune aging opens a new frontier in which pediatric and adolescent patients are not exceptions to the aging paradigm but key to understanding its pathological extremes. This reconceptualization holds promise for transforming how autoimmune diseases are detected, stratified, and treated across the lifespan.

## Data Availability

The original contributions presented in the study are included in the article/Supplementary Material, further inquiries can be directed to the corresponding author.
