# Supplementary material for: Inflammasome activation and accelerated immune aging in autoimmune disorders
Source: Front Aging. 2025 Sep 30;6:1688060. doi: 10.3389/fragi.2025.1688060 (PMC12517589; doi:10.3389/fragi.2025.1688060)
Supplement: Supplementary file 1 [file Table1.pdf]

**Supplementary Table 1: Emerging Therapeutic Approaches Targeting Inflammasome-Senescence Axis**

| Strategy | Example | Target Pathway | Mechanism of Action | Clinical Potential |
|---|---|---|---|---|
| **Senolytics** | Quercetin, Dasatinib, Fisetin | Anti-apoptotic Bcl-2 family proteins | Selective elimination of senescent cells | Reduces SASP burden; may restore tissue function |
| **SASP Modulators** | NF-κB inhibitors, JAK inhibitors (e.g., Ruxolitinib) | NF-κB, JAK/STAT signaling | Suppress inflammatory secretome without killing cells | May reduce inflammation without compromising immune surveillance |
| **NLRP3 Inflammasome Inhibitors** | MCC950, OLT1177 (Dapansutrile) | NLRP3, ASC speck formation | Prevent inflammasome assembly and cytokine release | Effective in multiple autoimmune/inflammatory models |
| **IL-1/IL-18 Blockade** | Anakinra (IL-1Ra), GSK1070806 (anti-IL-18) | IL-1β, IL-18 signaling | Neutralize cytokines downstream of inflammasomes | Interrupts feed-forward inflammatory loops |
| **Autophagy Enhancers** | Rapamycin, Spermidine, Trehalose | mTOR inhibition, lysosomal clearance | Restore cellular homeostasis, reduce inflammasome activation | Delays immune aging by promoting organelle recycling |
| **Epigenetic Reprogrammers** | DNMT inhibitors (e.g., Decitabine), HDAC inhibitors | DNA methylation, histone acetylation | Reverse senescence-associated epigenetic marks | Rejuvenates immune cell function and diversity |
| **Mitochondrial Function Enhancers** | NAD+ precursors (e.g., NR, NMN), CoQ10, Metformin | Mitochondrial biogenesis, ROS reduction | Improve energy metabolism, reduce mtDNA stress | Breaks the loop between metabolic dysfunction and inflammasome activation |
| **Caloric Restriction Mimetics** | Resveratrol, Metformin, 2-deoxyglucose | AMPK, SIRT1, glycolysis inhibition | Mimic effects of caloric restriction on immune metabolism | Enhances longevity pathways and suppresses chronic inflammation |
