# Supplementary material for: Inflammasome activation and accelerated immune aging in autoimmune disorders
Source: Front Aging. 2025 Sep 30;6:1688060. doi: 10.3389/fragi.2025.1688060 (PMC12517589; doi:10.3389/fragi.2025.1688060)
Supplement: Supplementary file 2 [file Table2.pdf]

Supplementary Table 2: Biomarkers for Immune Aging and Inflammasome Activity

Biomarker	Target	Sample Type	Diagnostic Utility
Telomere length	T/B cells	Blood	Biological age
p16^INK4a / p21^CIP1	Senescent cells	PBMCs, biopsy	Senescence indicator
IL-1β / IL-6 / IL-18	Serum cytokines	Plasma/serum	Inflammaging index
Caspase-1 activity	Inflammasome	PBMCs	NLRP3 activation status
Mitochondrial ROS	Multiple cells	Flow cytometry	Oxidative stress indicator
